# Professor Caterina Pistarini, Secretary General of the World Federation for NeuroRehabilitation & EFNR Research and Education Affairs: Adapted Interview from the 12^th^ World Congress for NeuroRehabilitation (WCNR), Vienna, 2022

**DOI:** 10.25122/jml-2023-1007

**Published:** 2023-02

**Authors:** Stefana-Andrada Dobran, Alexandra Gherman

**Affiliations:** 1 RoNeuro Institute for Neurological Research and Diagnostic, Cluj-Napoca, Romania; 2 Sociology Department, Babes-Bolyai University, Cluj-Napoca, Romania

**Interviewer: Stefana-Andrada Dobran**

**Interviewee: Professor Caterina Pistarini** °

° Director of Scientific Institute of Rehabilitation ICS Maugeri, Genova, Italy

Director of Neurorehabilitation Department of Clinical and Research Institutes of Salvatore Maugeri Company

Professor of Physical and Rehabilitation Discipline, University of Genova, Italy

Past President of the Italian Society of Neurorehabilitation (SIRN)

Presidium Member of the European Federation of Neurorehabilitation Societies (EFNR)

Chair of WFNR Special Interest Group on Mild and Severe Brain Injury

Caterina Pistarini is a qualified Professor of neurology and her research interest covers multiple neurorehabilitation fields such as stroke, cranial trauma, spinal cord injury, robotic therapy, and ICF Classification Method. Professor Pistarini has technical skills and competencies regarding the definition and organizational protocols of care for patients with neurological disabilities. She is also involved, as a member of Italian Health Committees, in activities related to the definition of national guidelines for the rehabilitation care of patients with neurological disabilities. Professor Caterina Pistarini also acts as chair of national and international projects on the development of treatment protocols in the field of neurorehabilitation.

**S.A.D.:** Dear Professor Pistarini, we are here, in Vienna, for the 12^th^ World Congress for NeuroRehabilitation organised by the World Federation for NeuroRehabilitation. What is your first-hand opinion of the event so far, and have you participated in any previous editions?

**C.P.:** Thank you for the question! My impression is that it is a valuable scientific networking event. We had over 1,200 attendees on-site and this is a very good result, in particular after a period when congresses were organised online, the participation in attendance is important. We respected the standard of the WFNR in organising this congress, in the form of lectures, symposia, workshops, and teaching courses for young people. This is the third day of the congress and the impression is really good. It is not my first experience in WFNR, I participated at the congresses organised by the WFNR for many years and now, as general secretary. It is a pleasure for me to be here.

**S.A.D.:** What do you believe is the overarching theme of this year’s congress?

**C.P.:** To spread the culture of neuro-rehab all over the world. I wish this congress to be an opportunity for sharing opinions, and experiences and that this congress is fruitful for new relationships and spreading the culture of neurorehabilitation all over the world.

**S.A.D.:** From your perspective, what is the role of hybrid multidisciplinary events in developing neurorehabilitation research and practice, and which similar avenues do you believe are worth exploring?

**C.P.:** Well, hybrid events are very useful, in particular for specialists who can’t attend the congress in person due to problems related to travelling, business, work duties, and then, there is the possibility to merge on-site presentations, talk sessions, and to offer the chance to every person to assist the congress on line; it helps announcing the activities in neuro-rehab and to offer to specialists this possibility.

**S.A.D.:** What do you believe is the added value of machine learning methods for predicting a positive outcome in patients with consciousness disorders?

**C.P.:** This is one of my fields of competence and this is a very interesting question because it opens up the inquiry about the collection of big data. And machine learning methods offer the possibility to make algorithms between some characteristics of patients – demographic characteristics, the severity of lesions, instrumental data, and so on, and stay also in the acute setting, and collect data about complications, comorbidities, and the outcome of the patients. And [they give] the possibility to evaluate a prognostic outcome, at several times, during the pathway of care. Today we are able, for example, to connect the data about the severity of the injury, the demographic characteristics of patients, and the possibility of survival as related to the complications. Concerning the treatment and, in particular, rehabilitation treatment, we have stopped here because we don’t have much evidence about the treatment of patients with disorders of consciousness. We have some limited cases, some limited samples, and then, we have not been able until now to achieve definitive results and to collect these data about treatments and prognostic outcome of these patients, about the recovery from the severity of a disability.

**S.A.D.:** What do you believe are the most remarkable advancements in the therapy for disorders of consciousness?

**C.P.:** Undoubtedly, among the medical strategies, there is the use of some medication to improve arousal and awareness of these patients. And this could be a step forward concerning recommendations. We have to work on the use of pharmacological agents that can help the recovery of arousal or awareness. Other strategies concern the use of technologies for these patients. For example, transcranial direct-current stimulation (tDCS) and transcranial magnetic stimulation (TMS), or other robotic therapies, can be strategies to improve and to announce the recovery, in particular after the recovery of consciousness, when a patient overcomes a period of severity, improves, and in order to address the strategies and the goals of our treatments, we can use the new technologies also for this field of neurorehabilitation, also for these patients.

**S.A.D.:** Do you believe TBI-related neuropathic pain is sufficiently addressed in research? And, if not, why so?

**C.P.:** No, not so much. Concerning one first study, in particular on severe brain injury patients, about neuropathic pain we do not have enough data and it is not a sufficient study. For severe patients, we may believe that neuropathic pain can be due to the inability of the patient to communicate; his behaviour can mean that he does not feel pain. And that is not true. For other patients, for example for patients with mild traumatic brain injury, the accurate assessment of pain and the coexisting problems related to mood anxiety, depression, cognitive problems, or motor problems, have to be studied in depth because we need some guidelines regarding pain relief for these patients. Because it can last for months, years also.

**S.A.D.:** Alright, thank you very much!

**C.P.:** Thank you!

